# Prognostic value of CD66b positive tumor-infiltrating neutrophils in testicular germ cell tumor

**DOI:** 10.1186/s12885-016-2926-5

**Published:** 2016-11-18

**Authors:** Yuta Yamada, Tohru Nakagawa, Toru Sugihara, Takamasa Horiuchi, Uran Yoshizaki, Tetsuya Fujimura, Hiroshi Fukuhara, Tomohiko Urano, Kenichi Takayama, Satoshi Inoue, Haruki Kume, Yukio Homma

**Affiliations:** 1 Department of Urology, Graduate School of Medicine, The University of Tokyo, Bunkyo-ku, Tokyo, Japan; 2 Department of Geriatric Medicine and Anti-Aging Medicine, Graduate School of Medicine, The University of Tokyo, Bunkyo-ku, Tokyo, Japan; 3 Department of Functional Biogerontology, Tokyo Metropolitan Institute of Gerontology, Itabashi-ku, Tokyo, Japan

**Keywords:** Tumor-inflitrating neutrophil, Testicular cancer, CD66b, Neutrophil

## Abstract

**Background:**

Prognostic value of immune cells is not clear in testicular germ cell tumors (TGCTs). We aimed to investigate the prognostic value of tumor-infiltrating neutrophils in TGCTs.

**Methods:**

A total of 102 patients who underwent orchiectomy for TGCT were investigated for CD66b positive tumor-infiltrating neutrophils (CD66b + TINs). Immmunostaining for CD66b was performed in 102 sections as described. Clinicopathological parameters as well as cancer specific survival and overall survival were assessed for correlation with CD66b + TIN density.

**Results:**

High density group was significantly correlated with tumor diameter ≥ 10 cm, presence of nodal/distant metastasis, S stage, diagnosis of nonseminomatous germ cell tumor (NGCT), and presence of venous invasion (*p* = 0.0198, *p* < 0.0001, *p* = 0.0275, *p* = 0.0004, and *p* = 0.0287, respectively). It was also significantly associated with cancer-specific and overall survival (logrank *p* = 0.0036, and *p* = 0.0002, respectively). Multivariate analysis showed that increased CD66b + TIN was an independent prognostic factor for overall survival (*p* = 0.0095).

**Conclusions:**

Increased CD66b + TIN was significantly associated with presence of metastasis, S stage, and nonseminomatous germ cell tumor diagnosis. It was also an independent prognostic factor of overall survival in patients with TGCT.

**Electronic supplementary material:**

The online version of this article (doi:10.1186/s12885-016-2926-5) contains supplementary material, which is available to authorized users.

## Background

Inflammation is considered to play a significant role in tumor progression in many malignancies [1]. Tumors that produce various inflammatory cytokines recruit immune cells such as neutrophils, and activate them to favor tumor growth and progression [2]. Increased levels of neutrophils are observed both in peripheral bloods and tumor environment in various cancers [3–11]. However, to our knowledge, there has been no literature regarding association between neutrophils and testicular germ cell tumors (TGCTs).

The aim of this study was to investigate the prognostic value of tumor-infiltrating neutrophils (TINs) in TGCT. By assessing the intra-tumoral environment using immunohistochemistry, we considered that it would provide more direct information than parameters based on peripheral blood samples. Relationships between TIN and clinico-pathological parameters including prognosis in patients with TGCT are described in the present study.

## Methods

### Patient characteristics and tissue preparation

The study included 102 patients who underwent orchiectomy for TGCT at The Tokyo University Hospital between 1985 and 2008. Clinicopathological parameters were retrospectively investigated from clinical records. Peripheral blood white blood cell and neutrophil counts were not available in 22 and 36 patients, respectively. Tumors were restaged using the TNM 2009 staging system [12]. Patients with nodal and/or distant metastasis (30 cases) were classified according to the International Germ Cell Consensus Classification (IGCCC) [13]. No patients received chemotherapy or radiation before orchiectomy.

CD66b immunostaining was performed to evaluate intra-tumoral neutrophils, since CD66b is uniquely expressed by neutrophils and not expressed in monocytes or myeloid cells [14]. In addition, CD66b is a preferable marker of aggressiveness in cancer when compared with other markers such as myeloperoxidase [6]. Sections were available in all 102 patients for CD66b immunohistochemistry. Sections were obtained from the same tumor blocks used for routine pathological evaluation. Therefore, haematoxylin and eosin (H&E) stained sections were also available for reference regarding areas of tumors and vessels.

Informed consent was obtained from all individual participants included in the study. This study was approved by the Ethics Committee of the University of Tokyo Hospital (approval number #2283), and is in accordance with the Helsinki declaration.

### Immunohistochemistry

Immunohistochemistry for CD66b staining was performed by the streptavidin-biotin method as previously described [15]. Six-micrometer-thick sections were deparaffinized with 2 changes of xylene for 3 min each, then dehydrated using decreasing concentrations of ethanol, and rinsed in Tris-buffered saline (TBS). Antigen retrieval was carried out immersing the sections in citric acid buffer (2 mM citric acid and 9 mM trisodium citrate dehydrate, pH 6.0) and autoclaved at 121 °C for 10 min. After cooling period of 3 min, the sections were washed with TBS and blocked with endogenous peroxidase with 0.3% H_2_O_2_. The sections were then incubated in 10% bovine serum albumin (BSA) for 30 min. The slides were incubated overnight at 4 °C with a primary mouse antibody for CD66b which was diluted at 1:200 (#305102, Biolegend®, San Diego, USA). After the sections were washed in TBS, they were incubated with CSA-2 kit (DAKO, Carpinteria, CA, USA). The antigen-antibody complex was visualized with 3,3′-diaminobenzidine tetrachloride (DAB) solution (1 mM DAB, 50 mM Tris-HCL buffer, pH 7.6, and 0.006% H_2_O_2_). All sections were counter-stained by Carazzi’s hematoxylin for 60 s. For negative controls, normal mouse IgG was used instead of primary antibodies.

### Immunohistochemical assessment

The density of CD66b + TINs was assessed in immunostained sections as in previous literature [7, 16]. Stained cells with clear boundary and sufficient intensity were recognized as CD66b + neutrophils. Cells with blurry stains and unclear boundary were neglected. TINs were evaluated within the tumor, but not in the area showing necrosis or artifacts. TIN was counted from ten random microscopic fields (×200). TIN Counts of entire 102 sections were 14.8 ± 38.8 counts/microscopic field (mean ± SD), or median value of 1 (range 0 - 262). By using the receiver operating characteristic (ROC) curve analysis, cutoff value was selected from overall survival status from which the largest AUC was obtained (AUC = 0.80217, Additional file 1: Figure S1). Cutoff value was selected from the point which was closest to both maximum sensitivity and specificity (21.6 counts/microscopic field). Therefore, high and low TIN density was defined as counts ≤ 21 and counts > 21, respectively.

Two independent observers (YY and TN) evaluated the stained sections, blinded to the patients’ clinic-pathological details. The third observer (TF) determined the density (high or low) in case of disagreement between the 2 observers.

### Statistical analyses

We used the statistical software JMP ^®^Pro version 10.0.2 (©2012 SAS Institute Inc., Cary, CA, USA) for data analysis. All continuous variables did not show normal distribution, and therefore Wilcoxon rank-sum test was used to compare differences between continuous variables between low and high TIN density groups. Pearson’s chi-square test and Fisher’s test (used when frequency was under 5) was used in analyzing differences of categorical variables between low and high TIN density groups in Table 2 and Table 3. Log-rank test was performed to analyze the statistical difference of cancer-specific and overall survival in low and high density groups. Multiple regression model was used to identify associated factors of cancer specific and overall survival. Variables that were significantly associated in univariate survival analysis were included in the multivariate analysis. Since the hypothesis ‘high TIN density increases the risk of cancer specific and overall mortality’ was considered unilateral, *P* value of < 0.025 was considered to be statistically significant in analysis regarding survival and multiple regression models. *P* value < 0.05 was considered to be statistically significant in statistical analysis evaluating association between other variables.

## Results

Clinical characteristics of 102 patients with TGCT are presented in Table 1. Median value (interquartile range (IQR)) of patient age was 34 (26 - 40) years. Fifty-eight patients had pathological stage T1, and 44 had T2–T4. Median values (IQR) of LDH, βhCG, AFP levels were 240 (170 - 463) IU/ml, 1.7 (0 – 20.2) mIU/ml, and 5 (2 - 141) ng/ml, respectively. Eighty and sixty-eight patients had preoperative clinical records of white blood cell and neutrophil counts from peripheral blood samples, respectively. Median values (IQR) of white blood cell and neutrophil counts were 6800 (5900-8375) counts/μl and 4300 (3542-5937) counts/μl, respectively.

**Table 1:** Clinical characteristics of 102 patients with TGCT

Variables		Median (IQR) or number of cases (%)
Age (years)		34 (26 - 40)
LDH (IU/ml)		240 (170 - 463)
αFP (ng/ml)		5 (2 - 141)
βhCG (mIU/ml)		1.7 (0 – 20.2)
Peripheral blood white blood cell count (/μl)	(*n* = 80)	6800 (5900 - 8375)
Peripheral blood neutrophil count (/μl)	(*n* = 68)	4300 (3542 - 5937)
T stage	T1	58 (56.9)
	T2	29 (28.4)
	T3	13 (12.7)
	T4	2 (2.0)
N stage	N0	72 (70.6)
	N1	9 (8.8)
	N2	7 (6.9)
	N3	14 (13.7)
M stage	M0	92 (90.2)
	M1a	9 (8.8)
	M1b	1 (1.0)

*TGCT* testicular germ cell tumor, *IQR* interquartile range, *LDH* lactate dehydrogenase, *AFP* α feto protein, *βhCG* β human chorionic gonadotropin

**Fig. 1:**
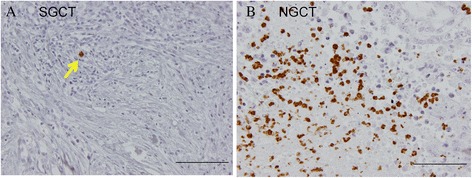
Representative examples of CD66b positive neutrophils in TGCT patients. Representative examples of CD66b + tumor-infiltrating neutrophils. Yellow arrow shows CD66b + neutrophil. Scale bars, 100 μm. **a** A patient with seminoma. **b** A patient with pure embryonal carcinoma. Abundant number of tumor-infiltrating neutrophil can be observed within the tumor

A total of 102 sections were examined for CD66b immunostaining. The number of intravascular neutrophils (Fig. 1) was considered negligible (mean count: 0.095 cells per microscopic field), and was not included in the TIN count. There were 81 cases for low, and 21 for high density group. There were no significant differences between high and low TIN density groups in terms of individual tumor markers and IGCCC risk (Table 2). TIN density did not show correlation with peripheral neutrophil counts (Wilcoxon rank-sum test *p* = 0.7947).

**Table 2:** Relationships between TIN density and clinical characteristics in TGCT patients (*N* = 102)

		TIN density		
		Low (*N* = 81)	High (*N* = 21)	*P* value
Age (years ± SD)^a^		34.7 ± 11.2	31.7 ± 12.3	0.1438
Tumor marker				
LDH (*N* = 99)	Normal	41 (42%)	7 (7%)	0.1768
	High	38 (38%)	13 (13%)	
AFP (*N* = 99)	Normal	55 (56%)	12 (12%)	0.2449
	High	23 (23%)	9 (9%)	
βhCG (*N* = 101)	Normal	31 (31%)	7 (7%)	0.6484
	High	49 (48%)	14 (14%)	
T stage	T1	50 (49%)	8 (8%)	0.0513
	T2-T4	31 (30%)	13 (13%)	
S stage (*N* = 97)	S0-1	58 (60%)	10 (10%)	0.0275
	S2-3	19 (20%)	10 (10%)	
Stage	Stage I	65 (64%)	7 (7%)	<0.0001
	Stage II-III	16 (15%)	14 (14%)	
IGCCC risk^b^	Good	10 (33%)	8 (27%)	0.8051
	Intermediate	4 (13%)	3 (10%)	
	Poor	2 (7%)	3 (10%)	

*TIN* tumor-infiltrating neutrophil, *TGCT* testicular germ cell tumor, *LDH* lactate dehydrogenase, *αFP* α feto protein, *βhCG* β human chorionic gonadotropin, *IGCCC* International Germ Cell Consensus Classification. ^a^Wilcoxon rank-sum test was used for statistical analysis in evaluating age between low and high density groups. Pearson’s chi square test was performed for other parameters. ^b^Note that the IGCCC risk classification is applied only in patients with metastatic TGCT patients

High density group was significantly correlated with diagnosis of NGCT (*p* = 0.0004), tumor diameter > 10 cm (*p* = 0.0198), and presence of venous invasion (*p* = 0.0287) (Table 3).

**Table 3:** Relationships between TIN density and pathological findings in TGCT patients (*N* = 102)

		TIN density		
		Low (*N* = 81)	High (*N* = 21)	*P* value
Pathology	SGCT	57 (56%)	6 (6%)	0.0004
	NGCT	24 (23%)	15 (15%)	
Tumor diameter (*N* = 91)	≤10 cm	69 (76%)	16 (18%)	0.0198
	>10 cm	2 (2%)	4 (4%)	
Tunica albuginea invasion	Absent	61 (60%)	12 (12%)	0.1001
	Present	20 (19%)	9 (9%)	
Venous invasion	Absent	62 (61%)	11 (11%)	0.0287
	Present	19 (18%)	10 (10%)	
Lymphatic vessel invasion	Absent	68 (67%)	17 (16%)	0.7473
	Present	13 (13%)	4 (4%)	
Tunica vaginalis invasion	Absent	72 (71%)	17 (16%)	0.4607
	Present	9 (9%)	4 (4%)	
Epididymis invasion (*N* = 100)	Absent	70 (70%)	19 (19%)	1.0000
	Present	9 (9%)	2 (2%)	
Spermatic cord invasion	Absent	72 (71%)	16 (16%)	0.1318
	Present	9 (9%)	5 (5%)	

*TIN* tumor-infiltrating neutrophil, *TGCT* testicular germ cell tumor. Pearson’s chi square test was used for statistical analysis except for ‘Tumor diameter’, ‘Lymphatic vessel invasion’, ‘Tunica vaginalis invasion’, and ‘Epididymis invasion’, which were analyzed by using Fisher’s test

**Fig. 2:**
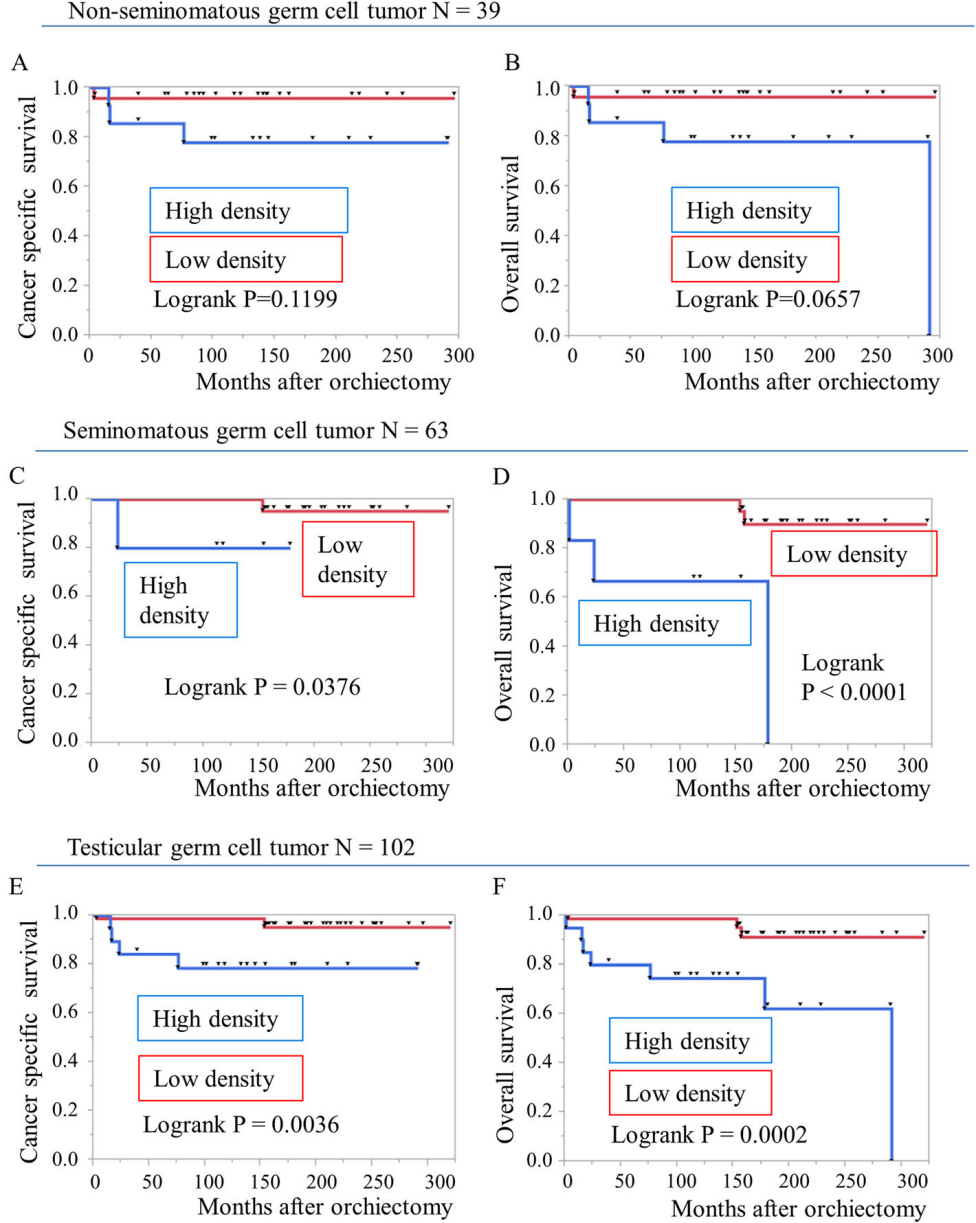
Survival analysis of CD66b + TIN density in testicular germ cell tumor patients. **a**, **b** Cancer specific and overall survival in patients with non-seminomatous germ cell tumor. **c**, **d** Cancer specific and overall survival in patients with seminomatous germ cell tumor. **e**, **f** Cancer specific and overall survival in patients with testicular germ cell tumor. Log rank test was used to analyze the differences in survival

Relationship of CD66b + TIN density to survival is shown in Fig. 2. High density group was significantly associated with poor survival for cancer-specific and overall survival in TGCT patients (logrank *p* = 0.0036, *p* = 0.0002, respectively). In addition, high TIN density group had lower cancer-specific and overall survival rates in SGCT patients (*P* = 0.0376 and *P* < 0.0001, respectively), whereas it showed tendency towards lower overall survival in NGCT patients (*P* = 0.0657).

In univariate analysis, clinical factors significantly associated with poor cancer specific and overall survival in TGCT patients were N stage (N1-3 vs N0), M stage (M1 vs M0), and TIN density status (High vs Low) (Table 4). In multivariate analysis, M stage was an independent factor of cancer-specific survival (*P* = 0.0126). High TIN density remained an independent prognostic factor of overall survival (*p* = 0.0095).

**Table 4:** Univariate and Multivariate analyses of risk factors predicting cancer specific and overall survival in patients with TGCT

		Cancer specific survival			Overall survival			
	Univariate analysis		Multivariate analysis		Univariate analysis		Multivariate analysis	
Risk factor	OR	*P* value	OR	*P* value	OR	*P* value	OR	*P* value
	(95% CI)		(95% CI)		(95% CI)		(95% CI)	
Age	5.67	0.0716			1.04	0.9479		
(< 34 vs ≥ 34)	(0.87-110.67)				(0.27-3.99)			
S stage	3.81	0.1518			2.02	0.3323		
(S2-3 vs S0-1)	(0.60-30.18)				(0.47-8.23)			
T stage	7.31	0.0364			3.47	0.071		
(T2-4 vs T1)	(1.12-142.8)				(0.90-16.89)			
N stage	14.20	0.0045	2.01	0.6487	7.00	0.0050	1.70	0.5818
(N1-3 vs ≥ N0)	(2.16-278.97)		(0.07-58.20)		(1.79-34.59)		(0.24-11.42)	
M stage	36.40	0.0002	16.10	0.0126	11.60	0.0034	4.94	0.1067
(M1 vs M0)	(5.78-315.97)		(1.76-375.69)		(2.37-56.96)		(0.71-40.97)	
TIN density	9.29	0.0112	4.107	0.1840	13.00	0.0003	8.17	0.0095
(High vs Low)	(1.68-71.07)		(0.51-40.67)		(3.21-66.21)		(1.67-47.73)	

*TGCT* testicular germ cell tumor, *OR* oddds ratio, *CI* confidence interval, *TIN* tumor-infiltrating neutrophil. Multiple regression model was used for statistical analyses. *P* value of < 0.025 was considered statistically significant

## Discussion

To our knowledge, this study is the first report to show that increased CD66b + TIN is an independent prognostic factor for overall survival in patients with TGCT. In addition to this finding, our results also revealed that increased CD66b + TIN was significantly associated with diagnosis of non-seminomatous germ cell tumor, S stage of S2 and over, tumor size > 10 cm, presence of nodal and/or distant metastasis, and presence of venous invasion.

In general, neutrophils are viewed as one of the first recruited effectors involved in acute inflammatory response [1, 14]. Neutrophils that are recruited to the tumor environment are discriminated from naϊve neutrophils, since they are characterized with low amounts of granules, and reactive oxygen species (ROS) [17]. They also express elevated levels of CXCL1, CXCL2 [17], that are potent chemoattractant promoting neutrophil recruitment [18]. Neutrophil recruitment is also supported by cancer cells that produce granulocyte colony stimulating factor (GCSF), which leads to an increment of neutophils via stimulation of bone marrow granulocytic cells [19]. In addition, tumor microenvironment stabilizes and prolongs the survival of neutrophils [18].

The function of neutrophils in tumor environment is complex, since they have conflicting function in cancer environment according to their activation state [17]. Tumor cells produce immunosuppressive transforming growth factor β (TGFβ) that promotes the polarization of tumor-associated neutrophils to a pro-tumoral “N2 phenotype” [20]. This type of neutrophil may contribute to cancer progression, since it produces growth factors such as vascular endothelial growth factor (VEGF), hepatocyte growth factor (HGF), MMP9, Bv8, and also have the capability to suppress cytotoxic lymphocytes [17, 18, 21, 22].

In this study, increased TINs correlated with presence of nodal and/or distant metastasis. This finding may be explained from the results of several studies that have identified the function of neutrophils to promote tumor migration and invasion. Head and neck squamous cell carcinoma (HNSCC) cells stimulated neutrophils to release proinflammatory cytokines which accelerated the migration of tumor cells [23]. Shamamian P et al. have shown that neutrophil serine proteases activated MMP2 via MT1-MMP, which lead to an invasion of fibrosarcoma cells [24].

In a clinical level, recent studies have shown that relationship between immune cells and tumor microenvironment is important in oncologic outcomes. In a study comprised of 121 patients undergoing nephrectomy for localized renal cell carcinoma, the presence of intra-tumoral neutrophils was an independent prognostic factor for cancer specific and overall survival [3]. In hepatocellular carcinoma patients, mean counts of intratumoral neutrophil were 27.3 ± 56.1 counts/microscopic field (×200), and presence of intratumoral neutrophil was a poor prognostic factor for hepatocellular carcinoma after resection [4]. Patients with low intratumoral neutrophils had a significantly longer 5-year recurrence free rate and overall survival (53% vs < 37% and 57% vs 18%, respectively). In colorectal cancer patients, increased intra-tumoral CD66b + neutrophil was not only positively correlated with pT status, M status, and clinical stage, but was also an independent prognostic factor in multivariate analysis [5]. Limitations include a possible bias in groups divided by TIN density because of the potential differences in comorbidity and treatment history.

## Conclusions

Increased CD66b + TIN was an independent prognostic factor for overall survival in TGCT patients. Thus, evaluating density of TINs may be beneficial as an additional prognostic tool. However, larger and prospective studies are necessary to further elucidate the present findings.

## Additional file

Additional file 1: Figure S1. Receiver operating characteristic (ROC) curve analysis was performed to determine the cutoff value of TIN counts by using the 0, 1 criterion. In the present study, overall survival status had the largest AUC (0.80217), and we selected cutoff value determined by overall survival status. Cutoff value was selected from the point which was closest to both maximum sensitivity and specificity (21.6 counts/microscopic field). Therefore, high and low TIN density was defined as counts ≤ 21 and counts > 21, respectively. (TIF 217 kb)

## Abbreviations

AFP: Alpha-feto-protein
IGCCC: International germ cell classification consensus
LDH: Lactate dehydrogenase
NGCT: Nonseminomatous germ cell tumor
TGCT: Testicular germ cell tumor
TIN: Tumor-infiltrating tumor
βhCG: Beta human chorionic gonadotropin
