# Supplementary figures and images for: Prognostic value of CD66b positive tumor-infiltrating neutrophils in testicular germ cell tumor
Source: BMC Cancer. 2016 Nov 18;16:898. doi: 10.1186/s12885-016-2926-5 (PMC5116192; doi:10.1186/s12885-016-2926-5)

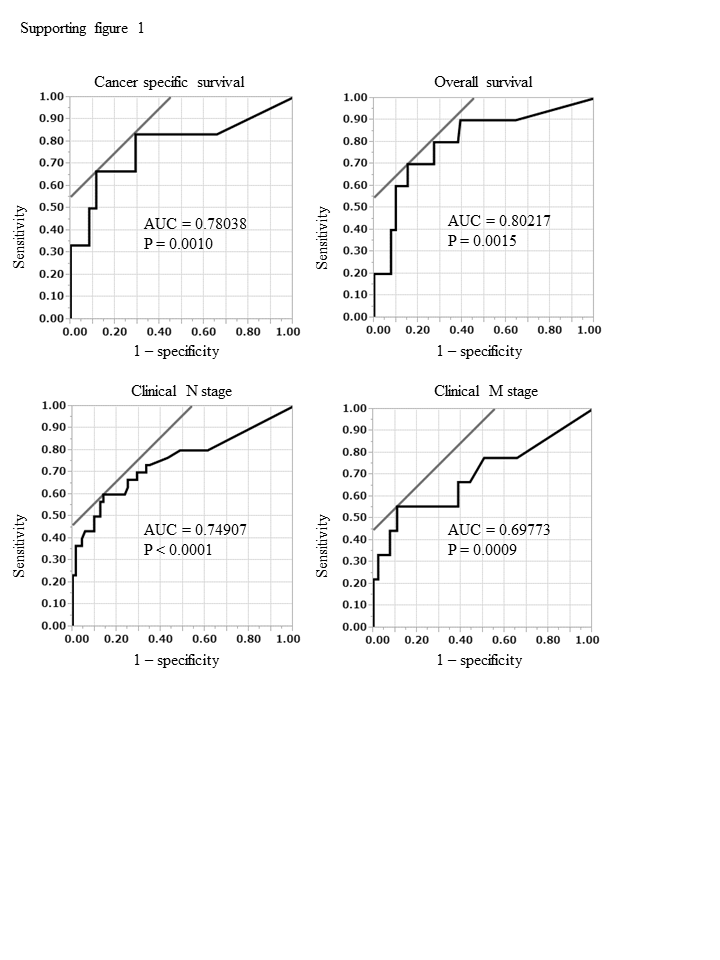

Supplement: Additional file 1: Figure S1. — Receiver operating characteristic (ROC) curve analysis was performed to determine the cutoff value of TIN counts by using the 0, 1 criterion. In the present study, overall survival status had the largest AUC (0.80217), and we selected cutoff value determined by overall survival status. Cutoff value was selected from the point which was closest to both maximum sensitivity and specificity (21.6 counts/microscopic field). Therefore, high and low TIN density was defined as counts ≤ 21 and counts > 21, respectively. (TIF 217 kb) [file 12885_2016_2926_MOESM1_ESM.tif]
